# Supplementary material for: Are carbon emissions trading and green financial instruments synergistic? -Comprehensive quantitative research based on content analysis
Source: PLoS One. 2024 Mar 7;19(3):e0298601. doi: 10.1371/journal.pone.0298601 (PMC10919601; doi:10.1371/journal.pone.0298601)
Supplement: S1 Data — (ZIP) [file pone.0298601.s001.zip › Data/地级市Z_F工作报告.zip!/╔╜╢1⁄2/┴─│╟╩╨/└┤╫╘╣1⁄2╓┌║┼í╛┬φ┐╦╩2╛▌═°í┐_2009.docx]

各位代表：

现在，我代表市人民政府向大会作工作报告，请予审议，并请各位政协委员和其他列席人员提出意见。

一、2008年工作回顾

刚刚过去的2008年是极不平凡的一年，是全市上下在挑战中把握机遇、在逆境中开拓前进的一年。一年来，我们坚持以邓小平理论和 “三个代表”重要思想为指导，深入贯彻落实科学发展观，认真执行中央、省委省政府和市委的决策部署，团结带领全市人民同心同德、顽强拼搏、扎实工作，全市的经济建设、政治建设、文化建设、社会建设和生态文明建设都取得了新的进展，顺利完成了市十五届人大一次会议确定的各项目标任务。

预计2008年，全市生产总值1200亿元，比上年增长13%；财政总收入119.9亿元，增长19.9%，其中地方财政收入48.9亿元，增长15.4%；全社会固定资产投资520亿元，增长25.5%；社会消费品零售总额393亿元，增长22%；城镇居民人均可支配收入14385元，增长16%，农民人均纯收入5041元，增长12%；年末城乡居民本外币储蓄余额572.2亿元，比年初增加120.7亿元。

一年来，我们重点抓了以下八个方面的工作：

（一）切实加大优质高效投入，经济发展后劲显著增强。始终坚持把中央宏观调控政策与聊城实际相结合，一方面着力消除经济运行中不健康、不稳定因素；另一方面，紧紧扭住科学发展、跨越发展不放松，坚定不移地上项目、上大项目、上好项目。全力抓好100个市级重点项目建设，完成投资220亿元，有63个项目建成投产。10月份以来，我们又抓住中央扩大内需的重大机遇，全力争取项目，中央新增的1000亿元投资中，我市争取了2.02亿元，列全省第七位。优质小麦生产基地续建项目得到国家批准，8个县（市、区）全部进入国家新增千亿斤粮食生产能力建设规划，京九铁路聊城段电气化改造、邯济铁路复线扩能改造、“四路一枢纽”等前期工作扎实推进。同时，着眼于长远发展，积极谋划了鲁西化工集团硅化工、莘县盐化工深度开发等一批战略性项目，已经取得重要进展。

（二）精心组织经济运行，促进经济平稳较快增长。始终坚持把又好又快发展摆在工作的首位。加大对农业的扶持力度，向农民发放各种补贴7.3亿元，投入农业基础设施建设资金2.8亿元，预计粮食总产459.9万吨、单产428.5公斤，均再创历史新高；油料、棉花、瓜菜总产分别增长7.5%、2.6%和11.6%；林业、牧业、渔业产值分别增长3.4%、16.5%和1.3%。面对资金、煤炭、电力紧张，原材料价格大幅波动等不利因素，积极做好协调保障工作，预计规模以上工业增加值、利税、利润分别增长17.5%、17%和15%。大力发展文化旅游、商贸物流等服务业，预计服务业增加值330亿元，增长15％，高于全市生产总值增幅2个百分点。

（三）大力推进结构调整，加快转变发展方式。预计三次产业比例调整为15∶59∶26。积极调整农业结构，林牧渔业产值占农业总产值比重达到23%；推进农业标准化，建成了市级农业综合检测中心，“三品”认证品种达到100个、基地面积184万亩；大力发展农业产业化经营，对重点龙头企业投入扶持资金1240万元，新增规模以上龙头企业21家，农民专业合作组织发展到595个，蔬菜、畜牧产业化取得明显成效。调整优化工业结构，利税过千万元的企业达到280户，其中过亿元的25户；有4种产品进入中国名牌产品国家专家组评审。实施高新技术创新示范工程，加强高新技术创业服务中心建设，新增工程技术研究中心11家、产学研合作创新基地27家，高新技术产业产值占规模以上工业比重提高1.5个百分点。在全省率先开展了生态文明市建设，创建国家环保模范城市工作通过了省级验收，节能减排成效显著，预计万元生产总值能耗比上年下降6.2%，二氧化硫、化学需氧量排放均削减5%，林木覆盖率达到29.2%。

（四）切实加强城市和新农村建设，为经济社会发展提供坚实支撑。坚持高水平规划，投资3000多万元，完成了古城区保护与整治、徒骇河城区段两岸控制等一批重大规划。坚持高标准建设，城市开发建设完成投资45亿元，其中聊城城区22.5亿元。以“两城一河”为重点的十大工程建设取得重大进展，东昌湖风景区完善、运河四期、旅游接待中心和古城门角楼工程建设进展顺利，体育公园场馆主体工程基本完工。围绕完善城市功能、方便居民生活，对柳园路等7条道路进行了改造，为15条小街巷安装了路灯，新建桥梁6座。实施绿化提升工程，新增城市绿化面积384公顷，城市绿化覆盖率达到38.8%。各县（市）城区建设也都取得了重大进展。加大对新农村建设的投入，各级财政共投入资金5.9亿元，增长21%，乡镇驻地详细规划覆盖率达到16.5%，中心村建设规划覆盖率达到100%；建设农村公路1295公里，改造农村危桥209座，新建农村客运站42个；新打、修旧机井7231眼，新增10.3万人用上自来水；新增农村有线电视用户4.2万家、沼气用户2.7万家。

（五）大力推进改革开放，为经济社会发展不断注入生机和活力。加快国有资产管理体制和国有企业改革，国有资产监管体制逐步理顺；临清德能等3家企业实现捆绑上市或借壳上市。继续深化财税改革，预算管理制度、转移支付制度进一步完善，国库集中支付、“收支两条线”改革取得新进展。积极推进事业单位改革，实现了机构编制、人员管理规范化。大力扶持民营经济发展，预计主营业务收入、利税分别增长26%和24.3%。加大招商引资工作力度，实际到位招商资金156亿元，增长13.2%。努力扩大对外开放，外经外贸在逆境中迈上新台阶，预计实际利用外资1.2亿美元，增长20%；进出口总额30亿美元，增长74%，其中出口15亿美元，增长50%，各项指标增幅均居全省前列。

（六）把改善民生作为政府工作的出发点和落脚点，积极发展各项社会事业。在财政十分紧张的情况下，努力加大民生投入。用于社会保障和教育的支出分别增长27.7%、21.4%，向农民承诺的“十件实事”全面或超额完成。认真做好就业工作，新增城镇就业再就业9.35万人，城镇登记失业率为3.1%。城镇职工养老、医疗、工伤、失业保险覆盖面进一步扩大；城镇居民基本医疗保险、城乡医疗救助制度全面推开；城市低保补助标准和农村低保标准进一步提高；农村五保财政供养制度得到落实，中心敬老院建设任务全面完成，集中供养能力达70%以上，初步建立起覆盖城乡的社会保障网络。着力解决困难群众住房问题，完成经济适用住房和廉租住房投资8598万元，施工面积19.4万平方米，发放廉租住房租赁补贴155.5万元；为350户农村贫困残疾人建设了新居。大力发展教育事业，实施农村中小学建设项目62个，新建校舍8万多平方米，建成了覆盖全市农村中小学和教学点的现代远程教育网；对全市义务教育阶段学生免除了杂费，农村学生免除了课本费，建立起覆盖各类学校的资助体系；全市本专科共录取2.9万人，其中本科1.29万人；学前教育、高等教育和职业教育健康发展。加快建立覆盖城乡的基本卫生保健制度，新建改建城市社区卫生服务中心10处、社区卫生服务站50处；新农合参合率达到95%，财政补贴标准每人提高40元，149处农村社区卫生服务站全部建成。扎实做好人口与计划生育工作,合法生育率比全省平均水平高2个百分点,人口自然增长率为6.5‰。积极发展文化事业,文化信息资源共享工程稳步推进;农村公益电影放映工程基本完成,共放映电影8.7万场;成功举办了2008水城之秋休闲周等文化活动。加快发展体育事业,第十一届全运会聊城赛区筹备工作进展顺利,竞技体育水平进一步提高,群众性体育活动更加广泛深入。坚持正确导向,弘扬社会正气,深入开展群众性精神文明创建活动,全社会文明程度不断提高。国防动员、人民防空、民兵预备役工作扎实有效,新闻出版、广播电视、社会科学、外侨、对台、妇女儿童、残疾人、民族、宗教、史志、档案、油区、地震、气象等工作都取得新进展。

（七）全力做好支援四川灾区和奥运保障工作，努力促进社会和谐稳定。面对四川汶川特大地震灾害，大力发扬“一方有难、八方支援”的优良传统，迅速向灾区派遣救援队伍，开展伤员救治和疫情防治，抢修灾区道路；积极组织赈灾捐助活动，共捐款物1.2亿元。全力做好灾区居民过渡性住房安置和援建工作，1500套高考活动板房第一个通过山东省、四川省验收，对口帮扶的片口乡灾后重建规划在山东省第一个编制完成并通过专家验收，加固和重建住房1527户，春节前力争让全部灾民搬进永久性住房。为确保北京奥运会、残奥会及青岛奥帆赛成功举办，积极做好安全保卫、食品安全、空气质量等保障工作，圆满完成了各项任务。以“隐患治理年”为主线，加大安全生产整治力度，安全事故起数和死亡人数分别下降2.1%、2.4%。加强社会治安综合治理，依法打击各种犯罪行为，做好群众信访工作，保持了社会和谐稳定。

（八）不断加强政府自身建设，提高政府依法行政水平。积极推进“五五”普法工作，加强对公务员特别是领导干部的法制教育。建立了科学发展工作研究制度、人民建议征集制度，广泛听取各方面意见，推进政府决策科学化、民主化。深化行政审批制度改革，扎实推进行政服务中心建设和全国行政服务标准化试点，精减各种审批、收费78项，行政效率明显提高。强化对重点工作事项的督查落实。进一步规范政务公开，加强政府网站建设，认真办好市长公开电话，不断拓宽群众与政府沟通渠道。切实改进工作作风，制定了“3·10”会议制度，进一步精简会议、提高效能。健全社会性公共事件应急预案体系，应对突发公共事件的能力明显增强。加强廉政建设和反腐败斗争，健全监督制约和绩效评估机制，严肃查办了一批违法违纪案件。

各位代表，2008年，我们遇到的复杂形势和矛盾困难是前所未有的。经济社会发展中煤电油运及资金紧张等困难问题需要我们加强协调调度，四川汶川特大地震灾害需要我们大力支援，北京奥运盛会需要我们全力保障，百年罕见的国际金融危机冲击需要我们沉着应对。但是，我们聊城没有停下加快发展、科学发展、跨越发展的步伐。我们在困难的情况下，成功实现了“经济较快增长、价格逐步回稳、结构加快优化、民生进一步改善”的总体目标，这是非常不容易的。这是在中央和省委、省政府正确领导下，全市上下团结一心、共同奋斗的结果，是聊城人民勤劳智慧、不畏艰难、自强不息的充分体现。过去一年政府工作的每一项成绩，经济社会发展的每一项进步，都凝聚着全市人民的智慧和心血，得益于全市人民的理解和支持。在此，我代表市人民政府，向全市工人、农民、知识分子、各级干部和社会各阶层的建设者，向各民主党派、工商联、无党派人士和社会团体，向驻聊人民解放军、武警官兵、公安干警和中央、省驻聊单位，向关心和支持聊城发展的海内外朋友，向全市所有纳税人，表示衷心的感谢和崇高的敬意！

各位代表，在肯定成绩的同时，我们也清醒地看到，我市经济社会发展还存在不少困难和问题，政府工作也存在不少缺点和不足。主要是：国际金融危机的冲击日益显现，经济发展的难度增大；外出务工农民返乡较多，农民持续增收难度加大；部分行业和重点企业生产经营困难，中小企业资金紧张，节能减排压力较大；地方财政减收增支因素较多，收支矛盾更为突出；就业形势依然严峻，社会事业和社会保障等还难以满足群众的需要，少数群众生活仍比较困难；一些不良社会风气仍然存在，社会稳定还潜伏着一些问题；有法不依、吃拿卡要的现象还时有发生，发展环境有待进一步改善。这些问题，无论是国内外形势的影响、历史因素的形成，还是工作中的不足，我们都必须高度重视，采取有效措施，认真加以解决。

二、2009年面临的形势及政府工作的总体安排

2009年是新中国成立60周年，是推进“十一五”规划顺利实施的关键一年，也是近年来我市面临挑战最为严峻、机遇最为重大、对今后发展影响最为深远的一年。做好今年的政府工作，对于维护改革发展稳定大局，加快建设强市名城实现新跨越，具有十分重大的意义。

各位代表，综观今年经济社会发展，形势依然相当严峻。当前的国际金融危机历史罕见、冲击力极强、涉及范围很广，目前不仅本身尚未见底，而且对实体经济的影响正进一步加深，全球经济面临数十年来最严重的衰退，国内经济增速下滑，2009年我们可能面临更加困难的局面。对此，我们必须充分认识，始终保持清醒头脑，尽量把困难考虑得更充分一些，把对策考虑得更周密一些。但是，更要看到，在变化的形势中也有难得的发展机遇，在逆境中也有不少积极因素。从宏观层面看，我国仍处于重要的战略机遇期，经济社会发展的基本面没有改变。中央出台了一系列扩大内需的政策措施，有利于我们加大投资力度、加快项目建设；有利于以国内市场为主、与基础设施建设相关产业的发展。十七届三中全会对农村改革发展作出战略部署，将对新农村建设起到强有力的推动作用。长三角、珠三角等发达地区产业结构面临升级，国内产业梯度转移步伐加快，有利于我们扩大招商引资。国际金融危机形成的倒逼机制，有利于我们加快调整结构、转变发展方式；能源原材料和设备价格大幅下降，有利于企业降低成本、扩大投资。从微观层面看，我市经济的外向度相对较低，周边市场广阔，受国际金融危机的直接冲击较小；综合经济实力显著增强，拥有一批实力强大的重点骨干企业，有着应对各种风险和挑战的坚实基础；随着一批铁路、高速公路的建设改造，聊城作为全国重要的区域交通枢纽、连接山东和中西部地区 “桥头堡”的地位更加突出。特别是经过改革开放30年的洗礼，我市形成了一批经受各种考验的优秀企业家队伍、党政干部队伍、科技人才队伍，广大干部群众的机遇意识、创新意识、开放意识、攻坚克难意识有了很大提高，全市上下政通人和、人心思上、共谋发展，这是我们战胜困难、加快发展最根本的决定因素！实现全市经济社会持续平稳较快发展是完全有条件的！

2009年，政府工作的总体要求是：全面贯彻党的十七大和十七届三中全会精神，以邓小平理论和“三个代表”重要思想为指导，深入贯彻落实科学发展观，坚决执行中央、省委省政府和市委的决策部署，围绕加快建设强市名城实现新跨越，按照建设生态文明市的要求，坚持工业强市、三产兴市、三农稳市、城建靓市方针，以保持经济平稳较快发展为首要任务，以项目建设为突破口，着力做好扩大内需、增强投资、调整结构、改革开放、改善民生工作，努力实现科学发展取得新进展、社会事业迈出新步伐、城乡面貌发生新变化、改革开放实现新突破、人民生活得到新改善、社会和谐见到新成效，以优异成绩迎接建国60周年。经济社会发展的主要预期目标是：生产总值增长12%，地方财政收入增长10%，全社会固定资产投资增长22%，实际利用外资增长12%，外贸进出口增长30%，社会消费品零售总额增长15%，城镇居民人均可支配收入和农民人均纯收入增长8%，居民消费价格指数控制在4%左右，城镇登记失业率控制在4.6%以内。主要约束性指标是：万元生产总值能耗下降6.4%，二氧化硫和化学需氧量排放分别下降1.5%和4.5%，人口自然增长率控制在7‰以内。

各位代表，我们确定今年的预期目标，把握的原则是，积极作为，科学务实。虽然主要经济指标增幅高于全国、全省平均水平，但与前几年相比速度有所放缓。这是基于严峻的国内外形势，必须实事求是、科学合理、切实可行。但在工作中，我们一定要坚定信心、迎难而上，尽最大努力，争取最好的结果。

在工作指导上，我们要切实把握好以下五个方面：

——应对当前困难与推动长远发展相结合。既要积极主动地采取各种有效措施，应对挑战，勇闯难关；又要抓住中央扩大内需的机遇，突出工作重点，科学上项目，全力保增长，为长远发展夯实基础，把迎接挑战的过程变成营造发展新优势的过程。

——保持增长速度与提高质量效益相统一。紧紧围绕经济建设这个中心,始终做到聚精会神搞建设、一心一意谋发展,努力保持经济平稳较快增长。同时,更加自觉地贯彻落实科学发展观,突出抓好发展方式转变和结构调整,加强自主创新、节能降耗和生态环境保护,进一步提高经济发展的质量和水平。

——加强城市建设与推进新农村建设相兼顾。进一步加强城市建设,加快城镇化进程,为新型工业化提供支撑。同时,大力推进农村改革发展,切实打牢“三农”基础,努力形成城乡经济社会发展一体化新格局。

——深入推进改革与扩大对外开放相促进。继续解放思想,更新观念,积极推进关键环节和重点领域改革,全方位推进对内对外开放,努力增强经济发展的活力和动力。

——加快经济发展与保障改善民生相协调。坚持一手抓科学发展,一手抓改善民生,在保持经济发展的同时,着力解决涉及群众利益的难点热点问题,加快发展社会事业,努力做到发展为了人民、发展依靠人民、发展成果由人民共享。

各位代表,挑战前所未有,机遇也前所未有。我们坚信,有党中央的正确指引,有省委、省政府和市委的坚强领导,有全市人民的大力支持,只要我们坚定信心、正视困难,审时度势、科学决策,周密部署、扎实工作,就一定能变压力为动力、化挑战为机遇,继续推动经济社会又好又快发展;就一定能在建设强市名城实现新跨越的征程上迈出新的更大步伐!

三、切实扩大投资和消费,确保经济平稳较快增长

全面完成今年政府工作的各项目标,必须抓住关键、突出重点。保增长是今年经济工作的首要任务,根本途径就是要加大投资和消费,增强对经济增长的拉动力。

(一)加大投资力度和优化投资结构。把加大投资、加快发展、调整结构结合起来,着力加大优质高效投入,拉动经济增长,促进结构调整和发展方式转变。

集中力量抓好项目建设。抢抓机遇,迎难而上,大力开展“项目突破年”活动。要以支持 “三农”、改善民生、完善基础设施、促进结构优化、节能环保为重点,科学安排,扎实推进,努力实现项目建设的新突破。一是争取项目实现突破。根据国家确定的六大类投资方向,按照省政府 “四个一批”、 “八个重点”的要求,筛选一批投资规模大、有利于结构优化升级的重大项目,切实做好争取工作,力争每个县(市、区)有更多的项目列入国家和全省建设计划。二是市级重点项目建设实现突破。继续举全市之力,抓好100个重点项目建设。重点抓好国家优质小麦生产基地续建、位山等大型灌区续建配套和节水改造等 “三农”项目;鲁西化工基地二期、祥光铜业40万吨阴极铜二期、莘县神荣盐化工、时风集团半钢全钢子午轮胎、中冶银河30万吨轻型胶版印刷纸、泉林纸业80万吨有机肥、希杰四期核苷酸等工业产业升级项目;聊城物流中心、古城区保护性开发、运河四期、马颊河旅游度假村等服务业项目;中冶银河与泉林纸业污水处理等节能环保项目;京九铁路聊城段电气化改造、聊城火车站改扩建、邯济铁路扩能改造、泰聊铁路、临高高速、德商高速、泰聊高速、济聊一级公路等重大基础设施项目。三是企业技术改造项目实现突破。坚持技术改造项目与新上项目同等重视、同等支持、一同考核,充分用好增值税转型、进口设备免税等优惠政策,千方百计为企业技术改造创造条件,力争技改投入280亿元,增长20%。四是谋划新的优势产业项目实现突破。围绕有色金属加工、新能源汽车、硅化工、盐化工、煤化工等新兴产业,搞好项目论证和申报,加快培育新的经济增长点,形成聊城长远发展的支撑力量。

完善加大投资的内在机制。对国家下达的投资计划,要积极落实配套条件,强化跟踪管理,加快建设步伐。抓好重点项目储备,充实完善项目库。进一步加大招商引资力度,完善工作机制,扩大招商成果。充分调动全社会特别是各类企业投资的积极性,下大力启动和扩大民间投资。搭建多层次银企合作沟通平台,优化金融生态环境,引导金融机构扩大信贷投放、支持企业投资。切实做好企业上市工作,鼓励企业捆绑上市、借壳上市,争取2家以上企业实现上市。

(二)积极扩大消费需求。坚持把消费作为经济增长的重要拉动力,努力保持消费市场持续快速发展。一是进一步提高居民收入。落实好优抚对象补助标准和企业退休人员待遇政策,加大强农惠农政策扶持力度,逐步提高对低收入家庭的补贴和补助,努力增加居民收入。加强社会保障体系建设,改善居民消费预期,增强消费信心。二是努力改善消费环境。加强城市商贸流通设施建设,完善社区便民服务设施,满足居民便利消费、放心消费的需要。健全农村流通网络,加快实施 “万村千乡”市场工程,完善农村日用消费品和生产资料流通体系,力争 “农家店”在乡、村的覆盖率分别达到90%和70%。组织实施好“家电下乡”工程。三是积极培育消费热点。稳定发展住房消费和汽车消费,着力发展服务消费、旅游消费和节假日消费,全面扩大居民消费需求。

四、突出抓好工业经济,努力营造工业发展新优势

工业是全市经济的支柱,保增长的关键是工业。要充分利用金融危机造成的倒逼机制,强化工业结构调整,构建现代产业体系,营造发展新优势。

(一)精心组织工业运行,大力推动企业振兴。加强对重点行业和重点企业生产经营情况的监测调度,进一步完善煤电油运保障机制,全力组织好资金和原材料供应。鼓励企业加强营销网络和营销机制建设,积极参加国内外重要经贸洽谈活动,全力开拓国内外市场。大力推进企业技术创新、管理创新、制度创新,抓好对企业家的培训,努力提高企业的核心竞争力。要尽心竭力地为企业搞好服务,努力减轻企业税费负担,严厉查处干扰企业正常生产经营的行为,为企业发展创造良好环境。

(二)大力推进自主创新,推动工业结构优化升级。加大对技术创新投入的政策扶持,整合财政投入、企业投入,集中力量推动重大科技攻关,重点抓好东阿阿胶、中通客车、时风集团等企业的60项重点技术创新项目,在中药现代化、生物制药、新能源汽车和机电一体化等方面取得突破。大力发展高新技术产业,深入实施高新技术创新示范工程,培育高新技术创新基地,力争高新技术产业产值占规模以上工业比重达到26%。搞好优势产业发展规划,引导企业加大技改投入,加快传统产业升级改造和落后生产能力淘汰步伐。积极搭建科技发展平台,充分发挥高新技术创业服务中心、聊城软件园、科技专家工作站的带动作用,力争新增博士后工作站2家,新增国家级、省级技术中心2-4个。深入开展与西安交大等高校院所的交流合作,鼓励支持高校特别是聊城大学领办科技创业园区。实施人才强市战略,不断创新和完善人才工作机制,为经济社会发展提供人才智力支持。大力实施质量兴市和标准化战略,力争新增中国名牌产品2个,采用国际标准产品6个,100家工业企业通过质量管理体系认证。

(三)加大重点企业支持力度,积极培育新的发展优势。围绕有色金属及加工、汽车及机械制造、冶金板材、化工、纺织、造纸等支柱产业的前延后伸,积极发展关联产业,拉长产业链条,巩固发展产业集群。发挥资源优势,加快盐化工等产业的培植,尽快形成新的经济增长点。对大中型企业特别是 “5513”工程企业,要在资金、土地、政策等方面给予积极扶持,鼓励兼并重组,支持技术改造,努力把企业做大、做强、做稳、做到可持续发展。

(四)切实抓好节能减排,努力改善生态环境。一是抓好节能降耗。鼓励和扶持企业研发新技术、应用新设备,提高技术创新对节能降耗的贡献率。抓好源头控制,严格控制新上 “两高一资”项目,加快淘汰落后产能、设备和工艺。继续抓好电力、化工、有色金属、造纸、建材等重点领域和34户重点用能企业的节能降耗,争取34户企业节能47万吨标准煤。认真落实差别电价政策,严格执行超标准耗能加价制度。积极推行清洁生产,实施 “2320”工程,推动循环经济发展。二是做好减排工作。实施燃煤电厂脱硫设施再提高工程。推进海河流域、南水北调水污染防治项目建设。加快县(市)城区和工业园区污水处理厂建设,完善配套管网,提高城市污水集中处理率。推广工业节水技术,抓好重点排污企业中水回用工程建设和运行,巩固提高减排成效。加强水、大气和固体废物污染防治,保持良好的生态环境。

五、加快发展服务业,着力在商贸物流、文化旅游业发展上实现突破

把加快服务业发展作为保增长的重要内容,以商贸物流、文化旅游为重点,突出抓好10个集聚区、30个重点项目建设,努力提高服务业在国民经济中的比重。

(一)大力发展商贸物流业。立足聊城作为连接山东与中西部地区 “桥头堡”的战略定位,发挥产业优势,集中力量发展一批物流基地、物流企业、大型市场,努力把聊城建成大宗物资集散中心和区域性中心商贸城市。加快推进聊城物流中心、粮食物流基地的规划建设,积极引进大型物流运营企业和物流开发企业。大力扶持千千佳、正大、铁力等重点物流企业发展。继续抓好工业企业主辅分离,引导大型企业集团将物流从核心生产业务中剥离出来,使之向第三方物流发展。加快大型市场建设和改造,重点抓好临清烟店轴承市场改扩建、开发区大东钢管市场升级改造、香江大市场三期等工程,努力完善市场功能,增强对外辐射力。积极推行连锁经营、大型超市、电子配送等新型运作模式,提高流通现代化水平。

(二)突出发展文化旅游业。紧紧围绕打造 “江北水城·运河古都——生态聊城”,积极推动文化产业与旅游业相互促进、共同发展。加快古城区、东昌湖、古运河、马颊河旅游度假村等旅游景区的开发建设,努力打造一批旅游精品。鼓励发展购物步行街区、高星级宾馆饭店、高档次休闲娱乐场所,完善旅游产业体系。尽快启动聊城旅游一卡通。继续举办文化旅游节、水城之秋休闲周等节庆活动,努力办出特色,打造品牌。大力挖掘传统文化,吸收现代文化,推出一批具有聊城特色的文化精品,丰富旅游文化内涵。发挥水城优势,积极举办各种国际国内赛事,发展水上游乐项目,打造水上运动之城、游乐之城。加强宣传营销,搞好区域合作,努力巩固省内旅游市场,扩大京津旅游市场,真正使聊城成为济南乃至山东的西花园,北京、天津等大城市的休闲度假基地。

(三)积极发展房地产、金融保险等新兴服务业。注意把握房地产市场供求关系,增加保障性住房供给,减轻居民合理购买自住普通商品住房负担,强化对房地产业的政策扶持,促进其平稳健康发展。进一步加大对辖区内金融企业的支持力度,提高信贷质量,防范金融风险。大力吸引市外金融机构来聊设立分支机构,支持设立和引进创业类投资公司。积极开展小额贷款公司试点。大力发展信托投资、融资租赁等非银行金融机构,引导业绩优良、管理规范的企业集团设立财务公司。加快发展保险业和证券业,推进信息服务、社区服务、中介服务等服务业发展。

六、大力推进农村改革发展,加快新农村建设步伐

应对国际金融危机,最深厚的基础在农村。聊城作为农业大市,更应当把 “三农”作为重中之重,巩固和发展农业农村经济好形势,促进农民持续增收。

(一)大力推进改革创新。按照统筹城乡发展要求,着力在农村改革关键环节上取得突破,进一步放开搞活农村经济,优化农村发展外部环境。一是规范农村土地管理。认真做好农村土地突出问题专项治理工作,规范完善第二轮延包合同,保持现有土地承包关系稳定并长久不变。建立健全土地流转市场,加强土地承包经营权流转管理和服务。完善征地补偿机制,保障农民合法权益,解决好被征地农民就业、住房、社会保障等问题。二是加强对农业的支持保护。严格执行国家惠农政策,加大各级财政对农业的投入,提高政府土地出让收益、耕地占用税新增收入用于农业的比例。抓好支农资金整合,提高资金使用效益。做好农民负担监督管理工作。加大扶贫开发力度,加快贫困区域群众脱贫步伐。加强农村信用体系建设,引导更多的信贷资金和社会资金投向农村。积极搞好农业政策性保险试点。三是建立促进城乡一体化发展机制。积极推进城乡规划、产业布局、基础设施建设、公共服务一体化进程,推动城乡经济社会发展融合。四是抓好其他各项改革。积极推行农村小型基础设施产权和集体林权制度改革,建立完善农村公路、自来水等基础设施养护管理体制。进一步深化强弱乡镇改革,激发乡镇发展活力。

(二)积极发展现代农业。按照高产、优质、高效、生态、安全的要求,加快构建现代农业产业体系,打响聊城无公害农产品品牌,推动传统农业向现代农业、农业大市向农业强市转变。一是加强优质粮源基地建设。按照 “稳定总产、提高单产、扩大优质、促进转化”的思路,积极推进优质粮食规模化种植试点工作,实行单收单打,实现优质优价。二是调整优化农业结构。在确保粮食生产能力的基础上,大力发展蔬菜、食用菌、花卉等高效经济作物和林牧渔业,打造蔬菜、畜牧百亿产业。积极推进优势农产品区域化布局、规模化生产,着力培植一批特色农产品生产基地。加快农产品批发市场建设,支持农村运销组织和运销大户发展,推动农产品生产基地与中心城市批发市场、大型超市对接,为农业结构调整提供支持。三是大力发展农业产业化。加大对农业龙头企业的扶持力度,开展“金政助农”活动,落实龙头企业新建扩建项目贴息政策,力争新增规模以上龙头企业30家。扶持农民专业合作社快速发展,力争达到400家。四是加快推进农业标准化。强化农产品质量标准、检测和认证体系建设,力争新增国家级农业标准化示范区2个、 “三品”认证24个、基地认证20万亩。五是提高农业科技装备水平。大力开展新农村建设科技示范工程,研究开发、转化示范40项先进适用技术,培育、建设10个科技示范基地。深入推进科技特派员工程,加强 “三个100”标准化、规范化建设。实施农村人才培训工程、 “三支一扶”和 “一村一名大学生”工程,鼓励高校毕业生回乡创业。落实农机补贴政策,重点突破玉米收获和保护性耕作,加快农业机械化。

(三)加强农村基础设施和环境建设。抓好农田水利设施建设,加快中低产田改造;抓好灌区续建配套和节水改造工程,新打机井2000眼、修旧3000眼。进一步改善农村交通条件,新建改造农村公路600公里,改造农村危桥156座;建设农村客运站31个,全面完成农村客运站建设任务,实现村村通客车,逐步实现农村客车班车化。继续搞好农村通自来水工程,新增10万农村人口用上自来水,自来水通水率达到90%以上。加强农村电力通讯设施建设,搞好农村中低压线路改造;加快实施有线电视村村通工程,农村有线电视通村率达到96%,新增用户6万户。加快农村可再生能源的开发利用,推广农村户用沼气池2.5万个、太阳能热水器2.5万户。实施农村小康环保行动计划,治理农业面源污染,控制农膜污染。积极开展植树造林,抓好美国白蛾防控工作,全市林木覆盖率提高1个百分点。

七、进一步加强城乡建设,努力提高城镇化水平

城乡建设是保增长、保民生、促发展的重要载体。要继续加强中心城市建设,抓好县(市)城区和村镇建设,加快城镇化进程。

(一)推进城乡规划全覆盖。按照城乡一体化发展的要求,组织编制 《城市空间发展战略规划》。加快古城区、徒骇河两岸修建性详细规划编制工作,搞好火车站站前区规划设计。加大城区公共设施布点、道路绿化、地热综合开发利用等专项规划编制力度。积极做好县域城市化发展规划,推进村镇规划编制,乡镇驻地详规覆盖率达到30%。完善国土资源规划体系,完成全市城乡建设用地增减挂钩规划,做好土地利用总体规划、矿产资源总体规划的修编和报批工作。严格执行各类规划,依法维护规划的严肃性和权威性。

(二)加强中心城市建设。按照 “江北水城·运河古都——生态聊城”的城市定位,突出 “水系、古韵和世界精品元素”三大特色,着力打造城市精品,重点实施八大工程：一是古城区保护性开发。加强基础设施建设,完成门楼、角楼和部分城墙建设,改造四关大街、沿街商铺,恢复部分历史古迹,努力打造 “水中古城”品牌。二是运河四期完善工程。重点做好河道开挖、衬砌和绿化,推进沿岸景观开发建设,进一步完善湖河水系。三是旅游接待中心工程。按照五星级标准完成整体项目建设,提高旅游接待水平。四是体育公园建设。在完成比赛场馆的基础上,启动全民健身活动中心等后续工程。五是聊城火车站改扩建工程。做好前期工作,争取年内开工建设。六是生态绿化工程。实施徒骇河截污、铃铛湖完善。抓好湖河绿化完善和主次干道、广场绿化的增量升级,推进立体绿化。七是环境综合整治工程。以商业街区为重点,对城市环境进行综合整治,改善市民生活居住环境。八是公用事业建设工程。重点抓好供热管网建设和改造、公共交通建设、管道燃气设施建设等项工作,城区新增集中供热面积30万平方米、管道燃气居民用户4000户。坚持高效能管理,进一步完善市政、环卫、绿化“三位一体”城市管理新模式;理顺城市管理行政执法体制,实现行政处罚权相对集中、行政执法工作全覆盖。巩固创建国家卫生城成果,积极创建国家环保模范城市、国家园林城市。

(三)抓好县(市)城区和村镇建设。各县(市)城区要重点抓好垃圾处理场建设、集中供暖供气、文化体育设施建设、水环境整治、城市绿化、城中村改造、背街小巷环境整治等项工作。加强中心镇建设,进一步完善功能,引导农村居民逐步向小城镇转移,加快城镇化进程。搞好新型农村住宅建设,引导使用新型住宅设计,推广节地、节能、节材新技术新材料。加强村镇环境综合整治,开展垃圾集中处理,不断改善农村卫生条件和人居环境。

八、继续深化改革开放,增强经济社会发展的活力和动力

应对国际金融危机,推动经济社会又好又快发展,必须坚定不移地推进改革,必须全方位扩大对内对外开放,努力增强经济发展的活力和动力。

(一)积极推进关键环节和重点领域改革。按照社会主义市场经济的改革方向,围绕消除经济社会发展的体制机制性障碍,进一步加大改革力度。

继续深化国有资产管理体制和国有企业改革。全面落实出资人职责,健全国有资产经营预算制度、国有资产保值增值考核办法以及重大决策失误和资产损失责任追究制度,完善国有资产监管运营体系,努力实现国有资产保值增值。继续深化国有企业改革,加快国有企业的整合重组,推动国有资本向重点行业、关键领域和优势企业集中;积极引进战略合作伙伴,推进国有企业股份制改革,实现投资主体多元化;加快劣势国有企业退出步伐,形成国有资本有进有退、合理流动的机制,推动国有经济优化升级。

大力发展民营经济。坚持把发展民营经济作为富民强市的重大举措,以 “平均每年每个乡镇增加民营经济税收300万元、固定资产过亿元”为目标,努力促进民营经济平稳较快发展。继续实施 “双百工程”和中小企业成长计划,选择一批规模大、前景好、水平高的民营企业进行重点培植,促使其迅速成长壮大。按照 “创办小企业、开发新岗位、以创业促就业”的要求,搞好小企业创业辅导基地建设,每个县 (市、区)要建成2处以上。实施群体加工业进村、进户计划,发展 “一村一品”、“一乡一业”。大力培植产业集群,抓好20个特色产业镇、10个中小企业服务平台建设,争取有5个镇、2个服务平台进入全省重点。加强信用担保体系建设,积极推动设立政策性担保机构,支持发展民营担保机构,鼓励组建各类会员制担保机构,确保每个县(市、区)担保机构达到2家。

加快财税体制和行政事业单位改革。积极探索财政支持经济发展的新方式,做好中央国债和城市建设债券的争取工作,适当扩大融资规模。依法加强非税收入征管,进一步压减收费项目,降低收费标准。积极稳妥地推进增值税转型。加快公共财政管理体制改革,深化部门预算、国库集中支付、 “收支两条线”、政府采购等项改革。切实做好财政增收节支,提高财政保障能力。进一步完善县乡财政体制,推进转移支付资金整合,促进县乡财政增收。搞好行政管理体制改革,加强公务员队伍建设。加快事业单位分类改革,完善事业单位人员聘用制度,严格落实事业单位公开招聘规定。

(二)全方位推进对内对外开放。对内对外开放是我市实现跨越发展的必由之路。面对严峻的国内外形势,我们必须立足于在困难中把握机遇,坚持 “引进来”与 “走出去”相结合,努力在应对危机中求得新的更大发展。

进一步加大招商引资力度。全年要实现招商引资到位资金180亿元,其中投资1000万元以上项目到位资金120亿元。继续巩固长三角、珠三角、日韩港台等重点招商区域,拓展胶东半岛和环渤海经济区,加大对欧美的工作力度,努力形成全方位、宽领域、多层次招商引资新格局。创新招商方式,全面实行产业招商,围绕重点产业积极组织重点企业和工业园区开展招商,进一步提高招商引资的规模和水平。有针对性地选择15家左右世界500强企业登门招商,力争在与世界500强合资合作上实现突破。积极引导现有大型外资企业增加投资。进一步加大对聊城经济开发区的扶持力度,加快工业新城建设,集中力量引进一批大项目和知名企业,努力使其成为全市高新技术产业的集聚区、新型工业化的示范区、全市经济的增长极。高标准搞好各县(市、区)开发区的规划建设,完善扶持政策,实行 “镇区合一、以区带村”的管理体制,推进与东部发达市开发区联动,努力加快发展步伐。

保持对外经贸稳定增长。一是优化出口结构。实施出口品牌战略,重点培育2-3个出口品牌。积极调整产品结构,努力增加自主性高技术产品、机电产品和高附加值产品的出口。引导企业利用国内外著名展会平台,建立自主营销网络,推动更多的企业进入联合国和跨国公司全球采购网。实施市场多元化战略,继续巩固韩日非欧等传统市场,积极开拓东盟、俄罗斯、新西兰、智利等新兴市场。二是积极扩大进口。抓住当前国际市场产品价格普遍降低的时机,积极帮助有关企业进口能源原材料产品,引进关键技术和先进设备。三是实施走出去战略。推动有条件的企业积极稳妥地对国外矿产、森林、农业等重要资源进行开发,搞好战略资源储备,提高企业可持续发展能力和市场竞争力。发挥聊建集团、中通钢构的示范作用,引导更多有条件的企业开展对外工程承包。加强外派基地县建设,提高外派劳务水平。充分发挥海关、商检、口岸的作用,为我市外经贸发展提供良好环境。

切实加强区域经济合作。加强同山东东部发达地区的合作,积极融入省会城市群经济圈,推进与济南一体化发展。以环渤海经济联合市长联席会为平台,全面加强与环渤海经济区,特别是北京、天津的合作。利用中原协作区、大京九经济合作带等平台,密切同中西部地区的联系,重点加强能源原材料合作,努力开拓中西部市场。

九、加快发展社会事业,切实保障和改善民生

保障和改善民生是政府工作的着力点,是保增长的出发点和落脚点。越是在困难的时候,越要高度关注民生,加快发展社会事业,努力为人民群众提供更多优良的公共服务产品,让广大群众安居乐业、共享发展成果。

(一)切实做好就业工作。就业是民生之本,事关社会稳定和经济发展大局。要进一步完善促进就业的小额担保贷款、就业援助等扶持政策,加强公共就业服务能力建设,组织好多种形式的就业促进和就业援助活动,确保全年新增城镇就业7万人。对生产经营遇到暂时困难的企业,鼓励采取灵活用工、弹性工时、组织培训等办法,尽量不裁员或少裁员,稳定现有就业岗位。继续打造劳务输出品牌,大力发展劳务派遣和境外劳务。积极帮助返乡农民工和高校毕业生以创业带动就业。统筹开展农村劳动力转移培训和返乡农民工就业、创业培训。切实维护劳动者的合法权益,建立和谐的劳动关系。

(二)加快完善城乡社会保障体系。完善城镇职工基本养老保险、基本医疗保险和失业、工伤、生育保险制度,扩大城镇居民基本医疗保险覆盖面,力争达到80%。加强城乡低保规范化建设,建立城乡最低生活保障标准与基本生活费用价格指数挂钩联动机制,保证低保标准自然增长。完善城乡医疗救助制度,逐步扩大覆盖面,提高救助水平。全面落实农村五保供养政策,健全供养经费保障机制,确保五保对象按标施保,进一步提高集中供养能力;开展农村社会养老保险试点。强化防灾救灾工作,提高灾害救助能力。积极推进安康居住工程建设。全市新建或改造廉租住房400套,建设经济适用住房10万平方米,免费为农村贫困残疾人建设新居1万平方米,为1528户城市低收入住房困难家庭提供廉租住房补贴保障。

(三)积极发展教育卫生等各项社会事业。优先发展教育事业。进一步完善义务教育管理体制,提高基础教育实施水平,保障进城务工农民子女与当地学生享受同等待遇。继续完善城乡中小学布局,组建、扩建城区中小学3所,新建、改建农村中小学55所。进一步改善农村办学条件,实施农村中小学仪器更新工程,开展 “211”工程试点。加强中小学教师队伍建设,扎实推进素质教育,努力提高教育质量。规范提高学前教育。积极发展民办教育,清理整顿义务教育阶段民办学校。大力发展职业教育,支持聊城职业技术学院发展,推进市直中等职业教育资源整合。进一步加强与聊城大学的地校合作。认真落实 “两免一补”政策和普通高中、职业学校、高等学校学生奖励资助政策。切实做好学校安全工作。加快发展卫生事业。完善农村三级卫生服务网络,改造乡镇卫生院10所,完成300所农村卫生室改造建设任务;新农合参合农民新增10万人,人均新农合基金增加到100元。抓好城市社区卫生服务体系建设,建立城市医院、预防保健机构与社区卫生服务机构分工协作机制。加强疾病预防控制,提高重大疾病防控和突发公共卫生事件应急处理能力。积极发展体育事业,大力开展全民健身活动,增强竞技体育实力,全力以赴搞好第十一届全运会聊城分赛区的各项筹备和办赛工作。坚持人口与计划生育工作基本国策,强化技术服务,规范流动人口管理,稳定低生育水平,促进人的全面发展。加强国防建设,深化军地合作,推动双拥工作向纵深发展,争创全省、全国双拥模范城 “三连冠”。认真做好对口援建工作,抓好片口乡8个项目建设。高度重视人口老龄化问题,积极发展老龄事业。关心支持妇女儿童、残疾人事业,进一步做好广播电视、新闻出版、社会科学、民族、宗教、对台、侨务、油区、人防、档案、史志、气象、测绘、防震减灾等各项工作。

(四)努力推动文化大发展大繁荣。以建设文化强市为目标,充分发挥聊城历史底蕴深厚、资源丰富的优势,大力发展文化事业和文化产业,努力把潜在的文化影响力转变为现实的城市竞争力,把丰富的文化资源转变为现实生产力,为加快建设强市名城实现新跨越提供强力支撑。一是加强公共文化建设。完成中国运河文化博物馆暨聊城市博物馆布展工作,建成乡镇综合文化站35个,文化信息资源共享工程社会覆盖面达到90%以上。广泛开展形式多样、丰富多彩的群众文化活动。积极做好国家级非物质文化遗产项目申报和文化遗产的挖掘保护利用工作,逐步实行国有博物馆、纪念馆免费开放。二是加快发展文化产业。积极培育传媒、出版、演艺娱乐、文化旅游等大型文化产业,扶持发展特色文化产业,培植引进文化创意、动漫设计等新兴文化产业,繁荣文化市场,推动文化消费,加快形成以重点文化产业为主导、相关产业联动发展的格局,建立起与我市经济社会发展相适应的文化产业体系。三是深入开展社会主义精神文明建设。把建设社会主义核心价值体系贯穿于文化建设全过程,不断增强贯彻落实科学发展观的自觉性和坚定性。围绕发扬聊城人文精神、培育生态文化、促进科学发展与社会和谐,深入开展文明社区、文明行业、文明窗口、文明单位等创建活动,为经济社会发展提供思想保证、舆论支持、文化条件。

(五)进一步加强民主法制建设和社会稳定工作。大力弘扬法治精神,营造政治文明、法制健全的良好环境,保障人民享有广泛的民主权利。积极推进社区建设,搞好村民自治,促进城乡基层民主政治建设。充分发挥民间组织的积极作用,增强社会自治功能。完善基层民主管理制度,进一步推行政务公开、厂务公开、村务公开。深入开展普法宣传,加强法律援助工作,提高全民法律意识和全社会的法制管理水平。高度重视社会治安工作,健全完善治安防控体系,依法打击各类刑事犯罪和境内外敌对势力的各种破坏活动,深入开展同法轮功等各类邪教组织的斗争。加大对经济领域犯罪的打击力度,保障经济正常运行。加强对重点群体的教育,做好预防青少年犯罪工作。深化维稳机制建设,强化矛盾纠纷排查化解工作,最大限度地预防、减少群体性事件的发生。正确处理人民内部矛盾,认真做好人民群众来信来访工作。牢固树立安全发展的理念,积极开展“安全标准年”活动,强化基层基础工作,严防发生各类重特大事故。切实抓好食品药品质量安全,加强医疗市场整顿,确保人民群众饮食用药就医安全有效。

十、全面加强政府自身建设,努力提高管理和服务水平

新的形势对政府工作提出了更新更高的要求。各级政府必须按照建设服务政府、责任政府、法治政府和廉洁政府的要求,以 “兴三风”为重点,全面加强自身建设,以良好的精神面貌和扎实的工作作风带领群众攻坚克难、共度时艰。

(一)大兴调查研究之风。坚持理论联系实际,把调查、学习、研究有机结合起来,努力增强把握下情的准确性、制定决策的科学性、出台措施的针对性,提高驾驭工作的能力。要深入实际、深入基层、深入群众,开展调查研究,做到问政于民、问需于民、问计于民。要切实加强学习,全力打造学习型机关、研究型队伍。要深入开展学习实践科学发展观活动,着力转变影响和制约科学发展的思想观念和体制机制。要加强对国内外形势和上级政策的分析研究,结合工作实际,提出切实可行的政策措施。要完善决策规则和程序,建立健全重大决策调查研究、社会公示、专家咨询、社会听证及责任追究制度,不断提高决策的科学化、民主化水平。各县(市、区)、经济开发区、市政府各部门和有关单位的领导班子全体成员,每年要安排不少于2个月的时间,带头深入基层,就聊城经济社会发展的重大问题进行调查研究,并完成高质量的调研报告。

(二)大兴干事创业之风。坚持高标准、严要求、快节奏、重成效,促使各级政府及其工作人员凝心聚力,真抓实干,创造新业绩、开创新局面。要切实抓好规划引导、组织协调、政策支持等关键环节,搞好基础设施、重点项目、民生项目、城市和新农村等项建设,努力推动经济社会发展。认真落实市委、市政府 《关于进一步优化发展环境的意见》和 《关于严格纪律强化问责切实转变作风的若干规定》,进一步深化行政审批制度改革,规范规费减免行为,实行企业公开评议市直部门及执法科室制度。加强电子政务建设,增强政府网上办事功能。要加强对社会事务的管理,完善突发公共事件应急管理体系,严格市场监管,保障和改善民生,推进基本公共服务均等化。要进一步树立艰苦创业的精神,坚持勤俭办一切事业,政府一般性开支和行政经费预算缩减5%。坚持 “既干事、又干净”,认真落实党风廉政建设责任制,加强对权力运作的监督制约,健全惩治和预防腐败体系,严格履行廉政承诺,保持清正廉洁,树立良好的公仆形象。

(三)大兴狠抓落实之风。坚持求真务实,在抓落实上狠下功夫,凡是定了的事情、看准了的事情,都要坚定不移地抓紧、抓实、抓好,切实抓出成效。要完善工作机制,实行政府部门目标责任管理,使各部门的工作具体化、数量化、项目化。要加大督查落实力度,加强对重点工作措施、进度、成效等方面情况的督查,激励多干事,褒扬干成事,治理不作为,惩戒乱作为。要切实减少各种评比达标活动,力戒形式主义、官僚主义。继续实行 “3·10”会议制度,进一步精简会议。要坚持依法行政,主动接受人大及其常委会的工作监督、法律监督和政协的民主监督,高度重视新闻舆论监督和社会公众监督,支持监察、审计部门依法履行监督职责,确保政府工作作风更加扎实、各项工作更加富有成效。

“同心促发展,奋力渡难关”。各位代表,全面完成今年政府工作的各项目标任务,最根本的,还是要靠全市人民共同奋斗。历史上,勤劳智慧的聊城人民百折不挠,战胜过无数艰难险阻;面对复杂多变的国内外形势,聊城人民也一定会不为任何风险所惧,不被任何干扰所惑,众志成城、奋力拼搏,创造更加美好的未来！让我们更加紧密地团结在以胡锦涛同志为总书记的党中央周围，高举中国特色社会主义伟大旗帜，坚持以邓小平理论和“三个代表”重要思想为指导，深入贯彻落实科学发展观，在省委、省政府和市委的坚强领导下，团结带领全市人民，努力开创我市经济社会发展的新局面，以优异成绩迎接建国60周年！
